# Evaluating the Effects of Polyphosphoric Acid (PPA) on the Anti-Ultraviolet Aging Properties of SBR-Modified Asphalt

**DOI:** 10.3390/ma16072784

**Published:** 2023-03-30

**Authors:** Yanling Xu, Kaimin Niu, Hongzhou Zhu, Ruipu Chen, Li Ou

**Affiliations:** 1 School of Civil Engineering, Chongqing Jiaotong University, Chongqing 400074, China; 2 Research Institute of Highway Ministry of Transport, Beijing 100088, China; 3 National and Local Joint Engineering Laboratory of Transportation and Civil Engineering Materials, Chongqing Jiaotong University, Chongqing 400074, China

**Keywords:** polyphosphoric acid, styrene butadiene rubber-modified asphalt, ultraviolet aging, rheological properties, modification mechanism

## Abstract

The ultraviolet (UV) aging of asphalt is an important factor affecting the long-term performance of asphalt pavement, especially in high altitude cold regions. The current studies have reported that styrene butadiene rubber-modified asphalt (SBRMA) has a good cracking resistance at low temperatures. In addition, polyphosphoric acid (PPA) is an effective modifier that can enhance the anti-UV aging properties of asphalt. However, the understanding of the improvement mechanism of PPA on the anti-aging of SBRMA remains unclear. Therefore, this study aimed to evaluate the effect of PPA on the UV aging resistance of SBRMA. The rheological properties of PEN90 asphalt(90#A), SBRMA, and PPA/SBR modified (PPA/SBR-MA) before and after UV aging were evaluated by dynamic shear rheometer (DSR) and bending beam rheometer (BBR) tests. The molecular weight and chemical structure of 90#A, SBRMA, and PPA/SBR-MA were determined by Fourier transform infrared spectroscopy (FTIR) and gel permeation chromatography (GPC), and the interaction and modification mechanism of the modifiers were analyzed. The rheological analysis shows that the high and low temperature performances of SBRMA are improved by adding PPA, and PPA also significantly reduces the sensitivity of SBRMA to UV aging. The microscopic test results show that PPA has a complex chemical reaction with SBRMA, which results in changes in its molecular structure. This condition enhances SBRMA with a more stable dispersion system, inhibits the degradation of the polymer macromolecules of the SBR modifier, and slows down the aging process of base asphalt. In general, PPA can significantly improve the anti-UV aging performance of SBRMA. The Pearson correlations between the aging indexes of the macro and micro properties are also significant. In summary, PPA/SBRMA material is more suitable for high altitude cold regions than SBRMA, which provides a reference for selecting and designing asphalt pavement materials in high altitude cold regions.

## 1. Introduction

Asphalt pavement is a major structure of highways globally because of its significant advantages, such as driving comfort, low noise, easy repair, and renewable nature [1,2]. After the asphalt pavement is opened to traffic, the surface asphalt is directly exposed to ultraviolet (UV) light, oxygen, water, and other factors in the external atmospheric environment. These factors lead to its continuous aging; that is, the chemical composition and microstructure of the asphalt are changed and will continue to deteriorate. Meanwhile, the repeated action under dynamic stress induced by traffic loading aggravates the aging of the asphalt concrete pavement, which is caused by climate factors including UV light and thermal oxidation [3,4,5]; this, in turn, leads to early diseases such as loose cracks and pits. Then, the durability of the asphalt concrete is seriously affected [6,7,8,9]. In the western region of China, the crack rate of asphalt concrete pavements is much higher than that in the inland region due to the low temperature and large temperature difference in the high-altitude area. The main reason for this is that the high-intensity UV radiation accelerates the photoaging process of asphalt materials in road applications. Studies have reported that UV radiation has a greater effect on asphalt cracking resistance at low temperatures than that of thermal oxidation and water aging [10]. Therefore, using asphalt material with good low temperature and UV aging properties is key to improving the durability of asphalt pavements in high altitude cold regions.

Since its discovery and use, the SBR-modified asphalt (SBRMA) has been well-known for its good low temperature performance. The adhesion between the asphalt and aggregate is enhanced because SBR can greatly improve the low temperature ductility and cohesion. Thus, asphalt pavements paved with this material has better crack resistance at low temperatures and the production method of SBR is also simple and low cost; consequently, SBRMA is widely used in cold areas [11]. In the high-cold and -altitude areas of western China, styrene butadiene styrene-modified asphalt, SBRMA, and rubber asphalt are commonly used, among which SBRMA is the most widely used type [12]. However, problems have gradually emerged with the increment in repealed loads and frequency, in which it lacks permanent deformation resistance and has seriously degrading anti-aging properties. Meanwhile, its poor storage stability seriously affects the use and development of SBRMA [13,14].

Many scholars have added polyphosphoric acid (PPA) modifier into SBRMA and improved the pavement performance of PPA/SBR-MA under synergy to overcome the defects of SBRMA. PPA can chemically modify asphalt. Specifically, a stable chemical bond is formed in the blending process of asphalt and the modifier, which enhances the compatibility and other properties. The current research indicates that PPA/SBR-MA has a better high temperature performance and fatigue resistance than base asphalt and SBRMA. The viscosity of asphalt is increased, the penetration degree is decreased, and the softening point is improved after the PPA modifier is added into SBRMA.

From the perspective of rheology, the high temperature rutting resistance of SBRMA is enhanced, the elastic modulus is significantly improved, and the phase angle is decreased with the addition of PPA [15]. The thermal storage stability and thermal oxygen aging resistance are also improved [16,17]. However, other studies have found that PPA gives SBRMA more light components and it becomes more sensitive to thermal and oxygen aging [18]. From the micro perspective, PPA is found to significantly improve the adhesion ability, high temperature elasticity, and rutting resistance of SBRMA through reverse flocculation; the incorporation of PPA also reduces the size of the polymer dispersed phase, which forms a more ideal microstructure and significantly enhances the compatibility of SBR and asphalt [19]. In regard to the mechanism of PPA improving the thermal oxygen aging resistance of asphalt, it has been suggested that PPA causes a gelation effect on asphalt and can disperse agglomerates of asphaltene micelles, which significantly reduces the impact of aging on the asphalt properties [20,21]. In addition, Han et al. [22] argued that a chemical reaction between PPA and the reactive groups in SBS-modified asphalt resulted in a tighter cross-linked network structure of the SBS-modified asphalt. Additionally, Ge et al. [23] suggested that PPA can ease the decomposition process and improve the stability of asphalt. Wei et al. [24] suggested that PPA can improve the aging resistance of asphalt primarily because it is not easily oxidized. However, studies on the effect of PPA on the low temperature cracking resistance of SBRMA have drawn different conclusions. Some scholars believe that PPA can reduce the low temperature cracking resistance of SBRMA [25], or that the low temperature cracking resistance first increases and then decreases with the rise in the PPA content [19]. Hao et al. [26] studied the cracking property of a PPA/SBR asphalt mixture at a low temperature through a thermal stress constrained sample test and semicircular bending test. They found that the low temperature cracking resistance of SBRMA can be effectively improved by the addition of PPA. In addition, PPA can also enhance the anti-UV aging properties of SBRMA [27].

In general, the addition of PPA can compensate for the deficiency of the permanent deformation resistance at high temperatures and the poor storage stability. However, the research about the anti-UV aging performance of asphalt by the addition of PPA was ignored. This study aimed to investigate the effect of PPA on the anti-UV aging behavior of SBRMA at the macro- and micro-scales, which determine whether PPA/SBRMA material is more suitable for high altitude cold regions than SBRMA. The anti-UV aging properties of PPA/SBR-MA, SBRMA, and 90#A were compared, and the excellent anti-UV aging properties of PPA/SBR –MA were analyzed from its chemical composition and molecular structure. Second, the effects of UV aging on the rheological properties were studied. Several anti-aging indexes were applied to compare the UV aging resistance of 90#A, SBRMA, and PPA/SBR-MA. The relationship between the changes in the chemical structure, molecular weight structure, and rheological parameters of the three asphalts in the UV aging process was further discussed.

## 2. Materials and Methods

### 2.1. Materials

In this study, the 90#A was obtained from the Karamay refinery of PetroChina. The basic technical indexes of 90A are shown in Table 1. The polymer modifier SBR was also purchased from a local company it is a white powder, and the specific technical specifications are shown in Table 2. The polyphosphoric acid (PPA) was purchased from a local supplier; its physical state is a viscous, colorless, and transparent liquid, and the basic technical indexes are shown in Table 3. 

### 2.2. Preparation of Asphalt

The manufacturer recommends that the reasonable dosage of PPA polyphosphate is 0.5–2.0 wt%, but the range is too large and has little significance for practical application guidance. Many domestic and international studies have shown that the appropriate dosage of PPA with SBS- and SBR-modified asphalt is 0.75–1.25% dosages by weight of the matrix asphalt [19,26,28]. In this study, an orthogonal test was used to select the optimal combination dosage and preparation process of PPA and SBR, and all the additive dosages are based on the matrix asphalt. The rate is generally 4000–4500 r/min to prevent the raw material reaching a temperature too high during shearing and to make the modified material mix shear homogeneous. The mixed shear process and the optimal mixing ratio were determined by orthogonal test after the PPA was added into the SBR asphalt binder. The optimum mixture ratio was 4 wt% SBR + 1 wt% PPA, and the mixed shear time for the PPA/SBR binder was 40 min. The temperature was 170 °C in the PPA/SBR-MA asphalt preparation process.

(1) Preparation of SBRMA

First, the 90#A was heated at 150 °C to a liquid state. Then, at a constant temperature of 160 °C, 4.0 wt% SBR modifier was continuously added to 90#A, and the blend was evenly stirred at a low speed for 15 min to make it fully swollen. Next, a high-speed shearing procedure (4000–4500 r/min) was performed for the blend at 165 °C for 40 min and stirred with a mixer for 30 min (speed of 600–700 r/min), followed by a curing procedure at 165 ℃ for 1 h to incubate constantly. Finally, the SBRMA was prepared.

(2) Preparation of PPA/SBR-MA

First, the 90#A was heated at 150 ℃ to liquid state. At a constant temperature of 160 ℃, 4.0 wt% SBR modifier was continuously added to 90#A, and the blend was evenly stirred at a low speed for 15 min to make it fully swollen. Next, a high-speed shearing procedure (4000–4500 r/min) was performed for the blend at 165 ℃ for 30 min. Then, the temperature was kept at 170 ℃. Thereafter, 1% PPA modifier was added to the asphalt binder for high-speed shear treatment, and the shearing rate was 4000–4500 r/min for 40 min. Stirring was performed at 160 °C for 30 min (600–700 r/min). Finally, the PPA/SBR-MA was prepared.

### 2.3. Testing Methods

**Figure 1:**
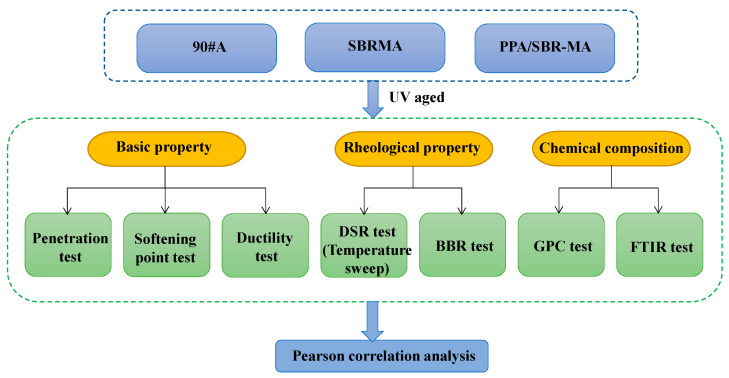
The designed process of experimental program.

Figure 1 shows the experimental framework of this study. The macro properties of the asphalt binder were characterized by a basic properties test and a rheological test. A GPC test and FTIR were performed to explain the mechanism and to characterize the microscopic aging effect.

#### 2.3.1. Ultraviolet (UV) Aging Procedure

**Figure 2:**
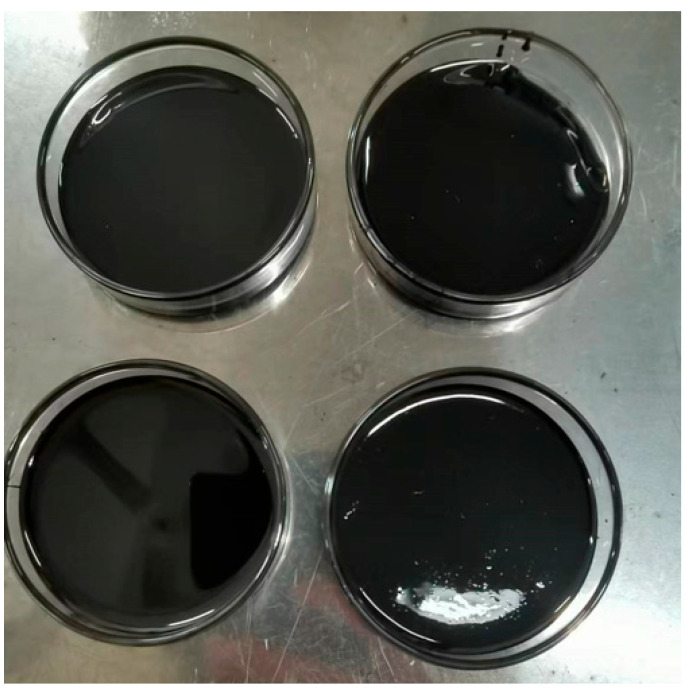
Asphalt before UV aging.

**Figure 3:**
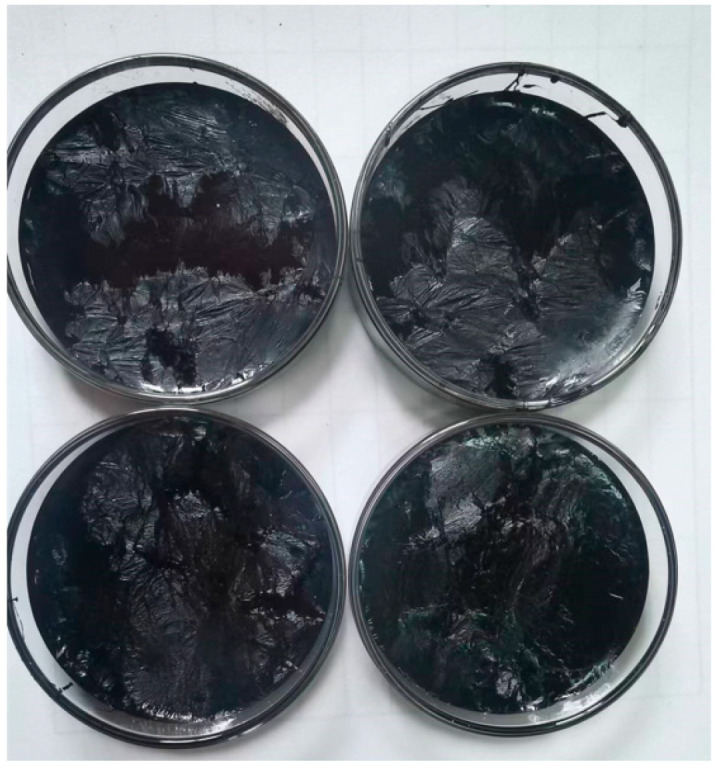
Asphalt after UV aging.

First, the density of each sample was measured for 90#A, SBRMA, and PPA/SBR-MA. Then, various unaged samples were uniformly spread on a steel plate (diameter of 140 mm). The thickness of each sample was controlled at 3 mm according to the relational calculation between the mass and volume. Some test samples are shown in Figure 2 and Figure 3.

The laboratory modeling UV aging experiment equipment of the asphalt was an opaque chamber. Two high-pressure lamb lights, with a light intensity of 500 W/m^2^, were installed on the top of the box. In the summer of the alpine region, the road temperature is often 30–40 °C higher than the air temperature and can reach 50–60 °C due to the unusually strong solar UV with the strong heat absorption ability of the asphalt mixture. Therefore, the UV aging chamber was kept ventilated and the constant temperature was 60 °C during the experiment. The radiation intensity of the sample surface in the simulated UV aging chamber was 75 W/m^2^ and measured by the light intensity instrument. The outdoor radiation intensity in this study was calculated based on the amount of solar radiation received in the Tibet region. Some studies have shown that the asphalt performance changes most obviously around 4 months [29]. Thus, an outdoor time of 4 months was selected to design the indoor UV radiation time. The indoor simulation time was 19.125 days based on the energy-equivalence of indoor and outdoor radiation. Thus, the indoor simulation time was 20 days.

#### 2.3.2. Standard Physical Properties Test

The basic properties of the three asphalts, including the penetration (25 °C, 5 s, 100 g), softening point, and ductility (5 mm/min, 5 °C), were conducted, respectively, in reference to the Chinese standard JTG-E20-2011 [30]. The basic properties aging indexes RP, SPI, and RD of the three asphalts were calculate by Equations (1)–(3) [31,32].

(1) $$\mathrm{RP}=\frac{{\;P}_{\;\mathrm{aged}}}{P_{\mathrm{virgin}}}\times 100\%$$

(2) $$\mathrm{SPI}=\frac{{\;(\mathrm{SP}}_{\mathrm{aged}}-{\mathrm{SP}}_{\mathrm{virgin}})}{{\mathrm{SP}}_{\mathrm{virgin}}}\times 100\%$$

(3) $$\mathrm{RD}=\frac{{\;D}_{\mathrm{aged}}}{D_{\mathrm{virgin}}}\times 100\%$$

#### 2.3.3. Temperature Sweep Test

The rheological properties of the three types of asphalt samples were characterized by the high temperature sweep test following the AASHTO-T315 method [33]. The type of dynamic shear rheometer used is DHR-2, manufactured by the America TA Instruments Company. The strain level is limited by 1.25%. The test temperatures were selected from 52 °C to 76 °C with the interval of 6 °C. The complex modulus $G^{*}$ and phase angle δ are recorded as viscoelastic parameters. The rutting factor aging index (RAI) at 64 °C was selected to evaluate the anti-UV aging performance of the different asphalts [34]. The RAI was calculated according to Equation (4). Notably, the anti-aging effect is better when the value is closer to 1.

(4) $$\mathrm{RAI}=\frac{(G^{*}/\sin\delta {)}_{\mathrm{aged}}}{(G^{*}/\sin\delta {)}_{\mathrm{virgin}}}$$

#### 2.3.4. Bending Beam Rheometer Test (BBR)

The BBR test was used to characterize the low temperature creep behavior of the asphalt binder, which was performed at −12 °C and −18 °C in accordance with AASHTO-T313 [35] in this study. In the test, the BBR was manufactured by the Superpave Company. The deformation was generated by a load of 980 ± 50 mN applied to the asphalt trabecular span. The average values of the flexural creep stiffness modulus (S) and the relaxation parameter (m) at 60 s were taken as the low temperature rheological parameters. They were used to analyze the low temperature rheological behaviors of the asphalt before and after UV aging. According to the studies by Li [36] and Xu [37], the ratio λ(λ = m/S) can be used to determine the low temperature performance when it is different between m and S. In general, the low temperature performance increases with the high λ value. Therefore, the ratio λ was determined in this study. The low temperature aging index (I_λ_) was used to quantify the influence of UV aging on the low temperature performance of the asphalt binder. The low temperature aging index I_λ_ of the three asphalts was calculated according to Equation (5). Notably, the anti-UV aging effect is better when the value is closer to 1.

(5) $$I_{\lambda }=\frac{\lambda_{\;\mathrm{aged}}}{\lambda_{\mathrm{virgin}}}$$

#### 2.3.5. Fourier Transform Infrared Spectroscopy (FTIR)

The FTIR test is an analytical tool used to characterize the different functional groups of chemical components. An IRprestge21 Fourier transform infrared spectrometer produced by Shimadzu Corporation of Japan was used to conduct infrared spectrum tests on 90#A, SBRMA, and PPA/SBR-MA before and after aging. The wave number ranges between 4000 and 400 cm^−1^, and the scan time was 32 times. 

The absorption peaks of C=O and S=O are approximately 1700 and 1030 cm^−1^, respectively, while the absorption peaks of the modified asphalt are about 966 cm^−1^ to identify the peak of SBR [38,39]. Some studies suggest that sulfoxides are also susceptible to additional oxidation to sulfones; then, the peak value of the sulfoxide group around 1030 cm^−1^ is not a reliable indicator of oxidative aging [38]. Meanwhile, the content of S element in Karamay based asphalt is 0. Thus, the sulfoxide group index was ignored in the quantitative analysis of the current study. After baseline correction, the ICO and BI indexes were computed as Equations (6) and (7) [40,41,42], and the aging index of the chemical structure (CR) was calculated according Equation (8) [4].

(6) $$ICO=\frac{\mathrm{area}\;\;\;\;\mathrm{around}\;\;\;1700\;\;\;cm^{-1}}{\mathrm{area}\;\;\;\;\mathrm{around}\;\;\;1460\;\;\;cm^{-1}+\mathrm{area}\;\;\;\mathrm{around}\;\;\;1375\;\;\;cm^{-1}}$$

(7) $$BI=\frac{\mathrm{area}\;\;\;\mathrm{around}\;\;\;966\;\;\;cm^{-1}}{\mathrm{area}\;\;\;\mathrm{around}\;\;\;1460\;\;\;cm^{-1}+\mathrm{area}\;\;\;\mathrm{around}\;\;\;1375\;\;\;cm^{-1}}$$

(8) $$CR=\frac{\mathrm{Index}\;\;of\;\;asphalt\;\;\mathrm{after}\;\;UV\;\;aged-Index\;\;\mathrm{of}\;\;asphalt\;\;before\;\;\;UV\;\;\mathrm{aged}}{\mathrm{Index}\;\;\mathrm{of}\;\;asphalt\;\;\mathrm{before}\;\;\mathrm{UV}\;\;\mathrm{aged}}$$

#### 2.3.6. Gel Permeation Chromatography Test (GPC)

**Figure 4:**
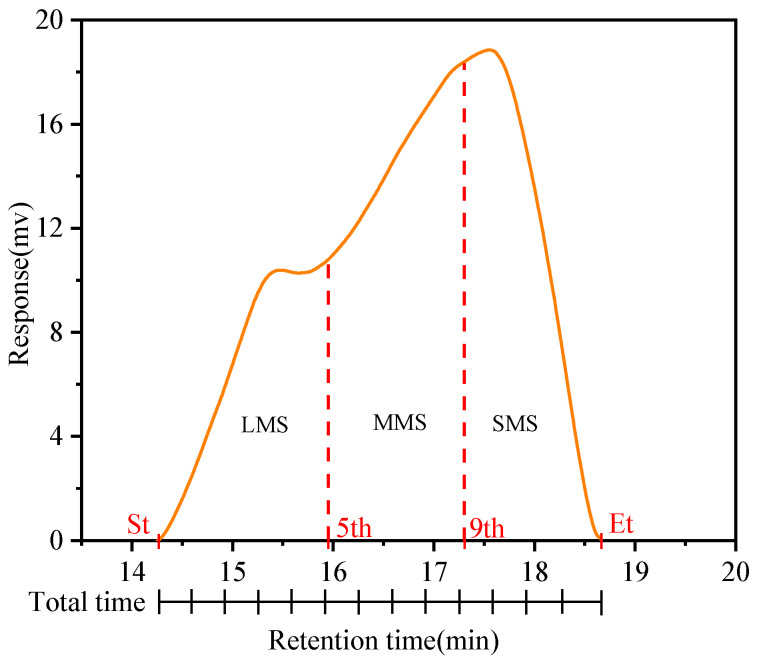
Methodology of analyzing molecular size.

Agilent 1260 GPC50 was adopted to determine the molecular weight distribution of the samples. In this study, tetrahydrofuran was used as the mobile phase solvent, and the solution passed through the columns at a flow rate of 1 mL/min. The columns were kept at 35 °C throughout the test, and the sample size was 100 μL each time. Polystyrene was made as the standard sample. Based on the theory of the GPC test, the large molecular size (LMS), medium molecular size (MMS), and small molecular size (SMS) were eluted successively with an increasing elution time. The chromatogram of each bitumen sample was divided into 13 sections according to the equal elution time between the beginning and end periods, with the first 5 sections representing LMS, sections 6 to 10 representing MMS, and the last 4 sections representing SMS [43,44,45]. The GPC curve is shown in Figure 4, and the three divided areas are expressed as percentages. The common average molecular weight includes the number average molecular weight (M_n_), weight average molecular weight (M_w_), and dispersion coefficient d. These parameters were calculated according to Equations (9)–(11) [46].

(9) $$M_{n}=\frac{\sum_{i=1}^{n}N_{i}\times M_{i}}{\sum_{i=1}^{n}N_{i}}$$

(10) $$M_{W}=\frac{\sum_{i=1}^{n}W_{i}\times M_{i}}{\sum_{i=1}^{n}W_{i}}$$

(11) $$d=\frac{M_{n}}{M_{W}}$$

where M_i_ is the molecular mass; N_i_ is the number of molecules of M_i_; W_i_ is the weight fraction of each type of molecule.

To further quantify the effect of UV aging on the molecular weight, the change rate of the molecular weight was used as the evaluation index, which was calculated according to Equations (12) and (13).

(12) $$I_{M_{n}}=\frac{{\left(M_{n}\right)}_{\;\mathrm{aged}}-{\left(M_{n}\right)}_{\mathrm{virgin}}}{{\left(M_{n}\right)}_{\mathrm{virgin}}}\times 100\%$$

(13) $$I_{M_{W}}=\frac{{\left(M_{W}\right)}_{\mathrm{aged}}-{\left(M_{W}\right)}_{\mathrm{virgin}}}{{\left(M_{W}\right)}_{\mathrm{virgin}}}\times 100\%$$

## 3. Results and Discussions

### 3.1. Standard Physical Properties

**Figure 5:**
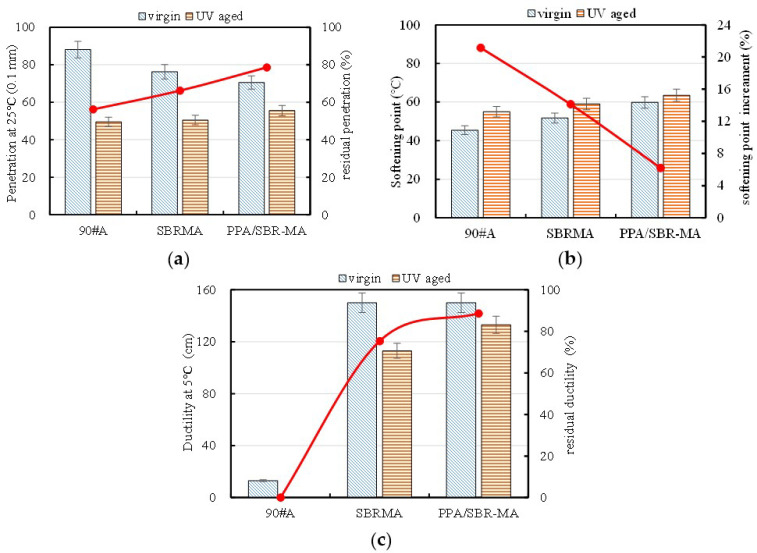
Basic property test results. (**a**) Penetration; (**b**) Softening point; (**c**) Ductility.

As shown in Figure 5a, the penetration of the three asphalts is decreased after UV aging compared to before UV aging. The penetration of PPA/SBR is obviously smaller than that of 90#A and SBRMA, which illustrates that PPA/SBR-MA has the highest hardness among the three asphalts. The sequence of the RP is PPA/SBR-MA, SBRMA,90#A, which shows that PPA/SBR-MA has the best UV aging resistance. The analysis results indicate that PPA can increase the hardness of SBRMA, but significantly reduces the sensitivity of the penetration to UV aging.

Figure 5b shows that the softening point of 90#A is the smallest, followed by that of SBRMA, and that of PPA/SBR-MA is the largest. Therefore, the addition of SBR can slightly increase the softening point of 90#A, while PPA can significantly increase the softening point of SBRMA and enhance its high temperature performance. After UV aging, the penetration of the three asphalts is decreased. The softening point of asphalt is increased because the oxidation of asphalt can increase the asphaltene. The sequence of the SPI is 90#A, SBRMA, PPA/SBR-MA, which shows that PPA/SBR-MA has the best UV-aging resistance among the three asphalts. PPA/SBR-MA has the best high temperature stability and the least influence of UV aging among the three asphalts. The analysis results show that PPA can improve the high temperature performance of SBRMA and reduce the effect of UV aging on it.

The ductility results of the three asphalts at 5 °C are shown in Figure 5c. Figure 5c indicates that the low temperature performance increases significantly with the addition of SBR, which is also the most important advantage of SBRMA. The ductility of SBRMA and PPA/SBR-MA are both greater than 150 cm (value equals 150 cm in calculation) before UV aging, which proves that the addition of PPA may enhance the low temperature performance or have no influence on the low temperature properties of SBRMA. The specific results will be further determined from the subsequent low temperature rheological test (BBR). The ductility of the three asphalts all decreased after UV aging. The RD was used to characterize the sensitivity of the low temperature flexibility on the UV aging. The sequence of the RD is PPA/SBR-MA > SBRMA > 90#A. The results show that PPA/SBRMA has a better low temperature flexibility than 90#A and SBRMA after UV aging, which indicates that PPA can significantly reduce the sensitivity of the low temperature flexibility of SBRMA to UV aging.

### 3.2. High Temperature Rheological Properties Analysis

#### 3.2.1. Phase Angle

**Figure 6:**
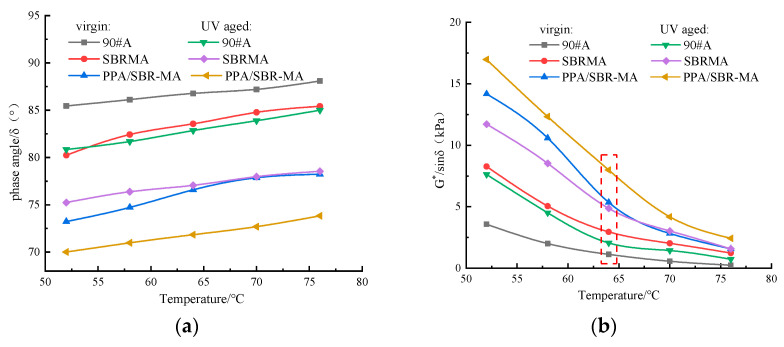

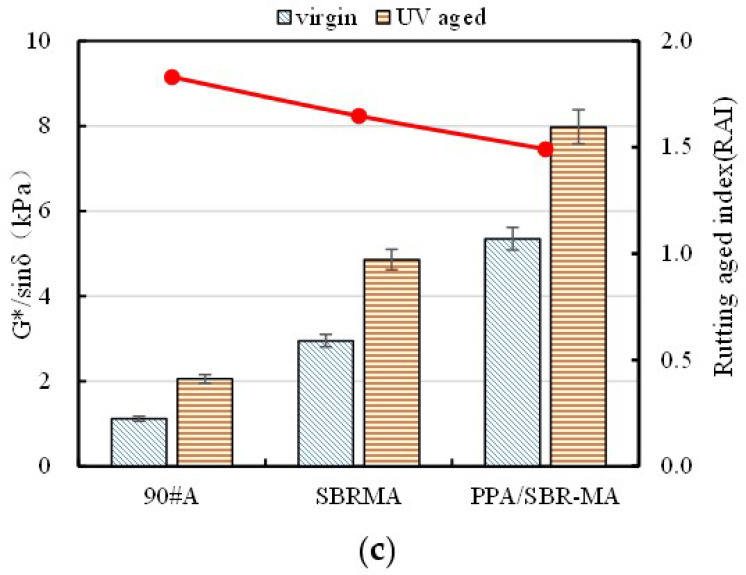
High temperature rheological properties results. (**a**) Phase angle of asphalt (δ); (**b**) Rutting factor ($G^{*}$/sinδ); (**c**) Rutting factor and aged index at 64 °C.

The phase angle mainly reflects the viscoelasticity of the asphalt binder. The elasticity and high temperature performance of asphalt are higher when the phase angle is smaller. As seen from Figure 6a, the phase angle increases with the rise in the test temperature. SBR and PPA contribute to the reduction in the phase angle. For example, at 64 °C, the phase angles δ of 90#A, SBRMA, and PPA/SBR-MA are 86.78°, 83.56°, and 76.59°, respectively. The phase angle δ of SBRMA is reduced by 3.7% compared with that of 90#A. The phase angle δ of PPA/SBR-MA is decreased by 11.7% compared with that of 90#A and 8.3% compared with that of SBRMA. Therefore, the results suggest that PPA enhances the high temperature performance of SBRMA.

The phase angle curves of the three asphalts all move downward after UV aging. The phase angles become smaller after UV aging, which indicates that the asphalts are hardened. The decreasing order of the values of the phase angle variable is SBRMA > PPA/SBR-MA > 90#A. For example, at 64 °C, the phase angles of 90#A, SBRMA, and PPA/SBR-MA before UV aging are 82.85°, 77.06°, and 71.84°, respectively. Meanwhile, the phase angles of the three asphalts are reduced by 4.5%, 7.8%, and 6.2%, respectively, after UV aging. The results indicate that SBRMA has a poorer anti-UV aging performance. The addition of PPA also reduces the sensitivity of the phase angle of SBRMA to UV aging.

#### 3.2.2. Rutting Factor

Figure 6b shows that the rutting factor decreases with the increasing temperature. When the test temperature is higher than 64 °C, the decreasing trend of the rutting factor of 90#A and SBRMA slows down. However, the decreasing trend of the rutting factor of PPA/SBR-MA slows down when the temperature is higher than 70 °C. The rutting factor of PPA/SBR-MA is obviously higher than those of SBRMA and 90#A. For example, at 64 °C, the rutting factor of SBRMA is increased by 163.4% compared with that of 90#A, and the rutting factor of PPA/SBR-MA is increased by 377.7% and 81.4% compared with those of 90#A and SBRMA, respectively. This result shows that PPA can obviously increase the high temperature performance of SBRMA and remedy the shortage of permanent deformation resistance. The rutting factor curves of the three asphalts move upward, and the rutting factor increases significantly after UV aging. The rutting factor shows a decreasing trend when the test temperature reaches 70 °C. Figure 6c shows the RAI of 90#A, SBRMA, and PPA/SBR-MA asphalts at 64 °C. The decreasing order of the RAI is 90#A > SBRMA> PPA/SBRMA. This result indicates that PPA/SBR-MA has the best high temperature stability under UV aging among the three asphalts.

In summary, the analysis results show that PPA can significantly improve the high temperature performance of SBRMA and reduce the effect of UV aging on it.

### 3.3. Low Temperature Rheological Properties Analysis

**Figure 7:**
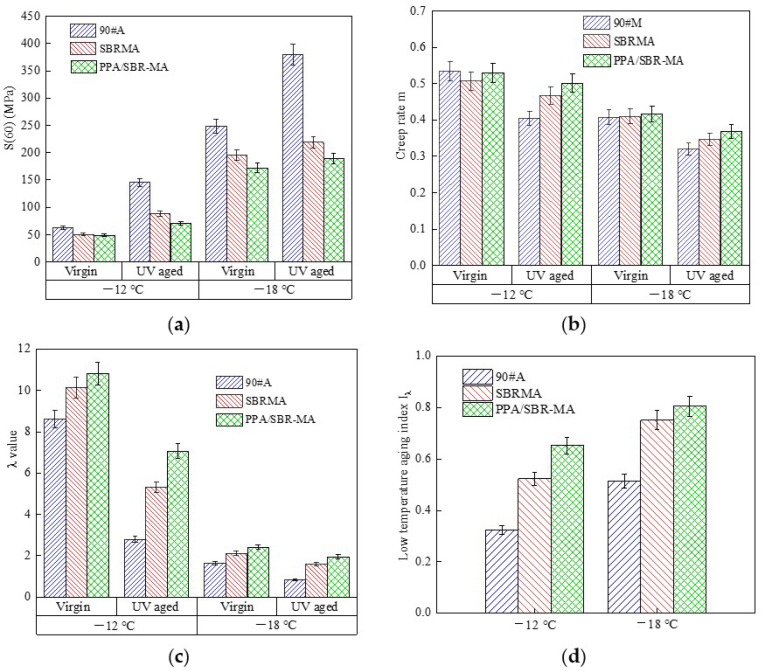
Low temperature rheological properties results. (**a**) Stiffness Modulus S(60); (**b**) Creep rate m; (**c**) The ratio λ; (**d**) Low temperature aging index.

Two test temperatures of −12 °C and −18 °C were used to test the 90#A, SBRMA, and PPA/SBRMA before and after UV aging. As shown in Figure 7a, the creep stiffness S(60) of 90#A decreases with the addition of SBR and PPA before UV aging, and S(60) of PPA/SBRMA is the minimum. The S(60) of the three asphalts are 61.9, 50.0, and 49.0 MPa, respectively, at −12 °C. Meanwhile, the values are 248.0, 194.5, and 172.0 Mpa at −18 °C. Therefore, the temperature stress of the asphalt increases significantly with the drop in temperature. The low temperature flexibility of SBRMA is better than 90#A, and the creep stiffness reduction rates of −12 °C and −18 °C are 19.2% and 21.6%, respectively. The flexibility of PPA/SBR-MA at low temperature is better than that of SBRMA, and the reduction rates of the creep stiffness at −12 °C and −18 °C are 2% and 11.6%, respectively. This result implies that the flexibility of PPA/SBR-MA is more obvious when the temperature is lower.

As shown in Figure 7b, the 90#A has a better in situ stress relaxation ability at −12 °C, and the m values of SBRMA and PPA/SBR-MA are equivalent to, or even slightly lower, than that of 90#A. At −18 °C, the advantage of the stress relaxation capacity of the modified asphalt gradually becomes obvious. With the addition of SBR and PPA, the m values of the modified asphalt increase successively to 0.406, 0.409, and 0.416. However, the increase is small. The results reflect that PPA can significantly improve the low temperature performance of SBRMA and reduce the sensitivity to UV aging. In addition, the lower the temperature, the more pronounced the effect.

Figure 7c shows that the λ value increases gradually with the addition of the SBR modifier and PPA, which indicates that the SBR modifier and PPA can enhance the low temperature performance of asphalt. The λ values of the three asphalts all decrease after UV aging. As displayed in Figure 7d, the aging index values of the low temperature properties at −12 °C and −18 °C are as follows: 90#A > SBRMA > PPA/SBR-MA. The above-mentioned analysis shows that PPA/SBR-MA has the best flexibility and stress relaxation ability before and after aging. The sensitivity of each parameter to UV aging also decreases with the decline in temperature, which is consistent with the reaction law of S(60) and m. The results show that the addition of PPA enhances the low temperature cracking resistance of SBRMA and reduces the sensitivity of UV aging. The effect of UV aging on the low temperature cracking resistance of asphalt can be better reflected by using the low temperature aging index I_λ_.

### 3.4. Chemical Structure Analysis

In this study, the molecular structure changes in the three asphalts were analyzed by FTIR test before and after UV aging, and the anti-UV aging mechanism of PPA/SBR-MA was explained.

**Figure 8:**
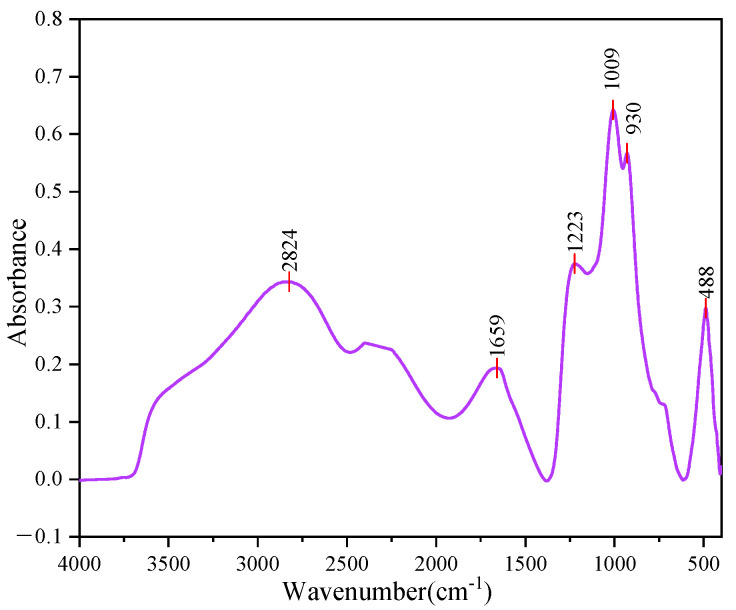
FTIR spectrum of. polyphosphoric acid (PPA).

**Figure 9:**
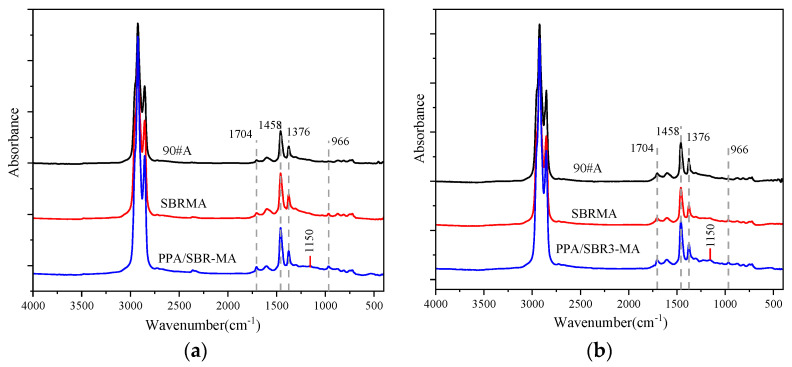
FTIR spectra of asphalt. (**a**) virgin; (**b**) UV aged.

Figure 8 shows the FTIR spectra of the PPA. Figure 9a shows that only marginal differences are found between 90#A and SBRMA. A new absorption peak of SBRMA and PPA/SBR-MA appears at 966 cm^−1^ because of the SBR characteristic peak vibration caused by the butadiene block vibration. Therefore, only physical interactions occur with the addition of SBR particles. This result agrees with the finding of a previous study [47]. A new absorption peak of PPA/SBR-MA appears at 1150 cm^−1^ compared with SBR-MA, which is mainly due to the stretching vibration of P–O and P=O [48,49]. However, the peak strength is low because the dosage of PPA is only 1%. As seen from Figure 8 and Figure 9a, the PPA infrared spectrum also shows the P=O stretching vibration frequency at 1223 cm^−1^, and the peak value disappears after PPA is added. Therefore, PPA chemically modifies the base asphalt [50,51], which is also an important reason why PPA can improve the compatibility of SBRMA. The characteristic peaks of PPA/SBR-MA and SBRMA either do not produce absorption peaks or the absorption peaks disappear. However, the intensity of the characteristic peaks changes. For example, the ICO and BI indexes are different after the addition of PPA, which indicates that the functional group of the asphalt binder does not change, but some chemical bonds change with the occurrence of chemical reactions [47]. Thus, whether chemical reactions occur between SBR and PPA needs further study.

**Figure 10:**
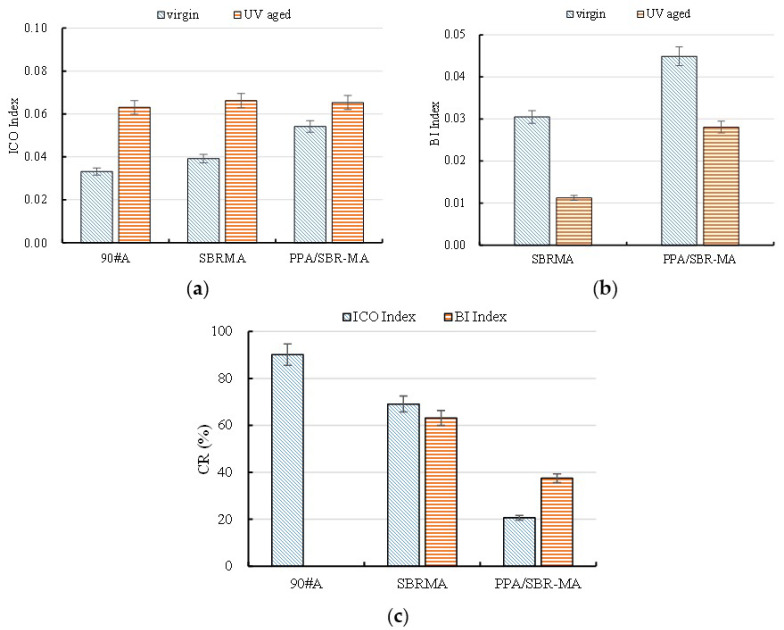
Chemical structure index of the three asphalts. (**a**) Carbonyl index; (**b**) SBR index; (**c**) Chemical structure aging indexes.

The influence of PPA on the anti-aging properties of the asphalt binders was determined by calculating the carbonyl group (ICO), SBR index (BI), and chemical structure aging index (RI) to quantitatively analyze the changes in the chemical bonds of the asphalt binders after UV aging. The analysis results of these indexes are shown in Figure 10. The carbonyl index (ICO) values of the three asphalts increase after UV aging, which is mainly caused by the oxygen absorption of the unsaturated carbon chain. The decreasing order of the ICO is 90#A > SBRMA > PPA/SBR-MA. Figure 10b shows that the BI index values of SBRMA and PPA/SBR-MA decrease after UV aging. After UV aging, the BI index value of SBRMA decreases from 0.031 to 0.011, while that of PPA/SBR-MA decreases from 0.045 to 0.028. The chemical structural aging index (CR) of PPA/SBR-MA is much smaller than those of 90#A and SBRMA, which indicates that the oxygen absorption of the saturated carbon chain and the degradation of the SBR modifier occur in the UV aging process. The addition of PPA effectively slows down the development and enhances the anti-UV aging property for SBRMA, which may be attributed to the complex chemical reaction between PPA and SBRMA. Thus, the results lead to the changes in the molecular structure, which effectively block polymer degradation and free radical diffusion. The reason for this is that the progression of UV aging is accompanied by the deep diffusion of free radicals and any delay in free radical diffusion can help delay the aging process [52].

### 3.5. Molecular Size Evolution Analyses

**Figure 11:**
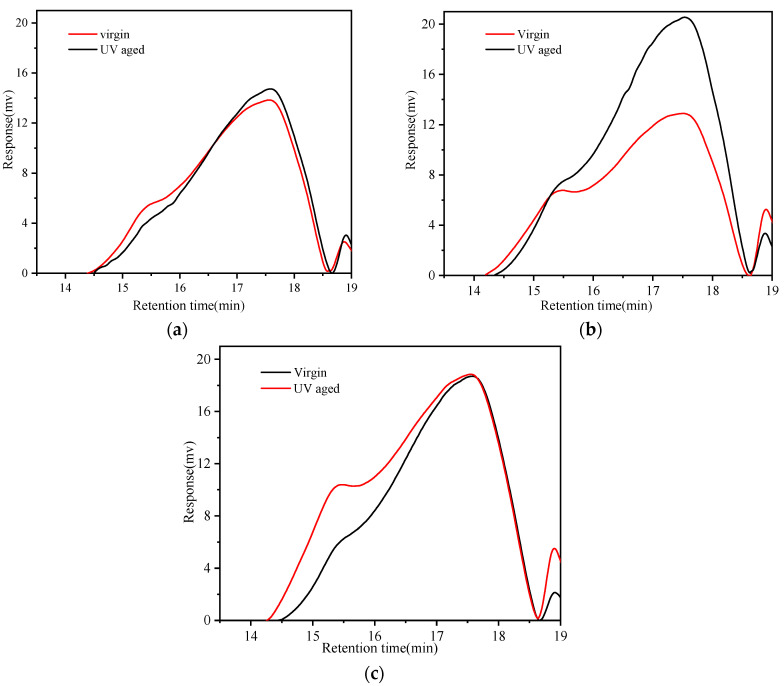
GPC curves of the three asphalts. (**a**) 90#A; (**b**) SBRMA; (**c**) PPA/SBR-MA.

Figure 11 shows that the addition of SBR significantly increases the macromolecules of 90#MA. Meanwhile, the addition of PPA results in a decrease in the LMS and an increase in the SMS, which implies that the addition of PPA transforms the heavyweight polymers to lightweight polymers [53]. The molecular weights of 90#A, SBRMA, and PPA/SBR-MA after UV aging decrease, which qualitatively reflects the changing trend of the molecular weights of asphalt before and after aging. The molecular weight distribution in the LMS range shows an obvious shoulder peak, and the percentage composition increases after UV aging. Therefore, UV aging would lead to intermolecular polymerization and promote an increase in the content of substances with large molecular weight. The molecular weight distribution of the three asphalts changes significantly after UV aging. The LMS values of the three asphalt cements are increased, but their MMS and SMS are decreased.

**Figure 12:**
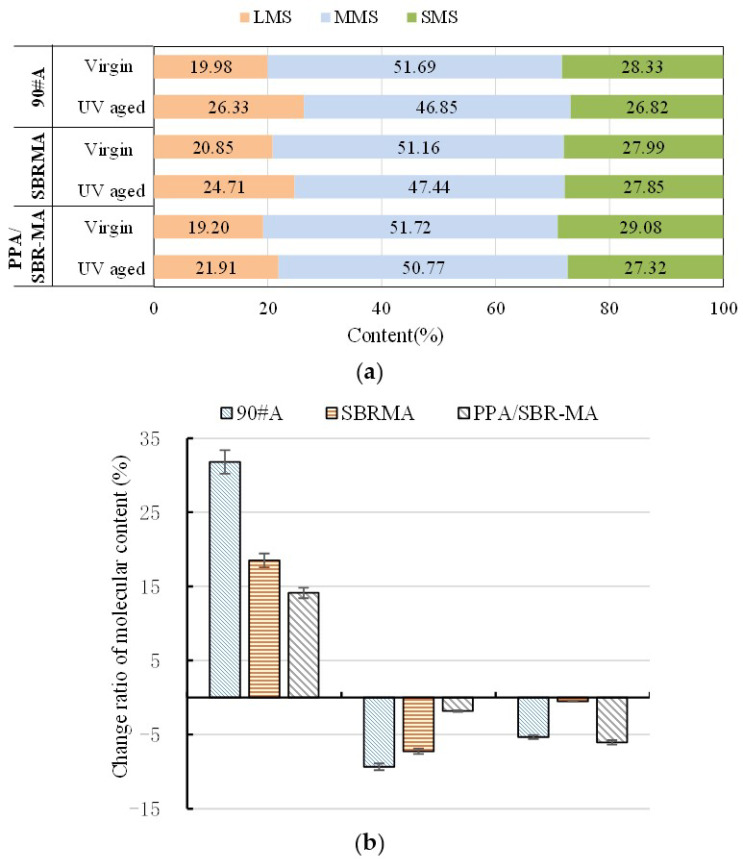
Molecular distribution analysis results. (**a**) Molecular distribution analysis results; (**b**) Change rate in molecular size.

Figure 12a,b show that the LMS increment of 90#A is the largest (31.79%), the LMS increment of PPA/SBR-MA is the smallest (14.13%), and the increment of LMS of SBRMA is in the middle (18.53%). Similarly, the increment of the MMS shows the same trend. The values are −9.36%, −7.27%, and −1.83% for 90#A, SBRMA, and PPA/SBR-MA, respectively. However, PPA/SBR-MA has the largest reduction in SMS (−6.08%), followed by 90#A (−5.33%), and SBRMA has the smallest (−0.52%). The reason is that 90#A may be primarily based on the transformation of the small and medium molecules to large molecules and the evaporation of small molecules in the UV aging process. Compared with 90#A, SBRMA improves the degradation of the SBR modifier polymer and increases the content of the medium molecule and small fraction. As a result, the change in the SMS is minimal. Furthermore, the addition of PPA inhibits the decomposition of the SBR polymer into small and medium molecules, and slows down the UV aging process of the base asphalt. Therefore, the conversion of medium molecules into large molecules is reduced, and the aging process is concentrated on the conversion of small molecules into medium molecules. This results in the smallest change in the LMS and MMS and the largest decrease in the SMS.

As shown in Table 4, the size rule of the parameters M_n_, M_w_, and d before UV aging is SBRMA >90#A > PPA/SBR-MA. The addition of the SBR modifier leads to a significant increase in heavy weight molecules, and the addition of PPA reduces the amount of SBRMA. The reason for this is that the addition of PPA induces a loss of hydrogen bonding and the decomposition of asphaltene, which result in a greater dispersion of smaller asphaltene domains [54]. The parameters M_n_, M_w_, and d of 90#A show an increasing trend after UV aging. Figure 12 also shows that the oxidation of components with medium and low molecular weights and the evaporation of components with low molecular weight mainly occur in the UV aging of base asphalt, which is consistent with the conclusions of previous studies [52,55]. The M_n_ value of SBRMA shows a decreasing trend after UV aging, but the Mw value shows the opposite trend. The main reason is that the polymerization of small and medium molecules and the degradation of large molecules of polymer-modified asphalt occur in the aging process, including the degradation of the polymer modifier. As a result, the polymerization of small and medium molecules increases the recombination fraction. The degradation of macromolecules and modifiers leads to the migration of the recombination to light components [56,57]. The change trend of M_n_ and M_w_ for PPA/SBR-MA after UV aging is consistent with that of SBRMA. However, the change rate of PPA/SBR-MA is significantly less than that of SBRMA. The addition of PPA significantly slows down the UV aging process of SBRMA and inhibits the degradation of the SBR modifier macromolecules. It greatly reduces the conversion of small to large molecules.

Comparing the molecular distribution and weight changes before and after aging proves that PPA gives the SBRMA a more stable dispersion system, inhibits the decomposition of the SBR modifier polymer, delays the aging process of base asphalt, improves the stability of SBRMA asphalt, and thus results in a better anti-UV aging performance.

### 3.6. Relationship Analysis of Microcosmic Property and Macroscopical Property

The Pearson correlation coefficient “r” of quantitative statistical analysis is often used in variable correlation analysis. The correlation between the macro and micro aging indexes were analyzed by the method in this study. As displayed in Table 5, with the exception of the micro aging index I_SMS_, the other parameters have good correlation with the macro aging index, which indicates that I_SMS_ is unsuitable for evaluating the aging effect and is only applicable to the mechanism explanation of the aging process. In particular, the correlation between I_LMS_, I_Mw_, and the macro performance index parameters is above 0.9, which implies that the distribution of large and average molecular weights has a good correlation with the macro performance. This deduction is consistent with previous research conclusions [43,47,58]. Thus, I_LMS_ and I_Mw_ can be used as the main evaluation indexes of the micro aging effect, and CR, CIO and I_Mn_ follow as the secondary indexes. The correlation analysis results of the macro and micro aging indexes suggest that the micro test can not only explain the micro action mechanism, but can also predict the macro performance.

## 4. Conclusions

The macro and micro properties of 90#A, SBRMA with 4% SBR modifier, and PPA/SBR-MA with 4 wt% SBR + 1 wt% PPA additives before and after UV aging were studied in this work. The key findings are as follows:

(1) The penetration of asphalt decreases with the addition of PPA, which proves that PPA/SBR-MA becomes harder and more resistant to deformation. The increase in the softening point indicates a higher temperature stability of asphalt. The incorporation of PPA significantly reduces the influence of UV aging on the consistency, high temperature stability, and low temperature flexibility of SBRMA according to the aging indexes of the penetration, softening point, and ductility.
(2) The addition of PPA significantly improves the high temperature deformation resistance of SBRMA before UV aging according to the analysis of the phase angle δ and rutting factor $G^{*}/\sin\delta$. The high temperature performance of the three asphalts is improved after UV aging. However, the rutting factor aging index (RAI) of 90#A, SBRMA, and PPA/SBR-MA are 1.83, 1.65, and 1.49, respectively, indicating that PPA can reduce the sensitivity of the high temperature rheological parameters of SBRMA to UV light.

(3) Based on the variations of S(60) and m before and after UV aging, it is found that UV aging will crack the low temperature properties of asphalt according to the change rule of these values. However, PPA/SBR-MA has the best flexibility and stress relaxation ability, which implies that PPA enhances the low temperature cracking resistance of SBRMA. The low temperature aging indexes I_λ_ of 90#A, SBRMA, and PPA/SBR-MA are 0.32, 0.52, and 0.65 at −12 °C temperature, and 0.51, 0.75, and 0.81 at −18 °C, respectively, which indicates that PPA can significantly reduce the UV aging sensitivity of SBRMA.
(4) The FTIR and GPC tests show that the addition of PPA gives the SBRMA a more stable dispersion system. UV aging mainly leads to the oxygen absorption of the saturated carbon chain and the degradation of the SBR modifier, along with the evaporation of small molecules. PPA can significantly enhance the anti-UV aging property of SBRMA because it mainly inhibits the degradation of the SBR modifier and delays the aging process of base asphalt.
(5) The microscopic aging indexes I_LMS_ and I_Mw_ after UV aging are significantly correlated with the macroscopic aging indexes RP, SPI, DR, RAI, and I_λ_ by the analysis of the Pearson correlation coefficient. They can be used as the main evaluation indexes of the microscopic aging effect. The ICO and I_Mn_ have a good correlation with the macro performance aging index. However, I_SMS_ is unsuitable for evaluating the aging effect and is only applicable to the mechanism explanation of the aging process. Microscopic experiments can not only explain the microscopic mechanism of action, but can also predict the macroscopic properties.

Overall, based on the results of the experimental data in this study, PPA can improve the high and low temperature performance and UV aging resistance of SBRMA, and PPA/SBR-MA is more suitable for high altitude cold regions than SBRMA.

## Figures and Tables

**Table 1:** Basic technical indexes of 90#A.

Parameter	Results
Penetration (25 °C, 0.1 mm)	88.1
Softening point (°C)	45.4
Ductility (15 °C, cm)	>150
C (%)	87.02
H (%)	9.31
O (%)	3.12
N (%)	0.51
S (%)	0

**Table 2:** Basic technical indexes of SBR.

Parameter	Results
Granularity (16-mesh, %)	98.6
Bound styrene content (%)	23.0
Mooney viscosity (ML)	55.5
Tensile strength (MPa)	25.0
Breaking elongation (%)	378
300% Constant tensile stress (MPa)	15.0

**Table 3:** Basic technical indexes of PPA.

Parameter	Results
Phosphoric acid (H_3_PO_4_, %)	115.2
Phosphorus pentoxide (P_2_O_5_, %)	83.5
Chloride (Cl, %)	<0.0003
Fe (%)	0.0014
As (%)	0.009
Heavy metal (Pb, %)	<0.002

**Table 4:** Results of molecular weight analysis.

Sample	Index				
	M_n_	M_w_	d	I_Mn_ (%)	I_Mw_ (%)
90#-virgin	909	3634	3.998	23.65	43.51
90#-UV aged	1124	5215	4.640		
SBR-virgin	998	3996	4.004	−4.81	24.27
SBR-UV aged	950	4966	5.227		
PPA/SBR-virgin	905	3460	3.823	−1.77	12.86
PPA/SBR-UV aged	889	3905	4.393		

**Table 5:** Pearson correlation results.

Macro-Aging Index	Micro-Aging Index					
	I_LMS_	I_MMS_	I_SMS_	I_Mn_	I_Mw_	CR_ICO_
RP	−0.9412	0.9823	−0.1848	−0.7754	−0.9783	−0.9870
SPI	−0.9505	0.9765	−0.1568	−0.7931	−0.9838	−0.9821
DR	−0.9949	0.8052	0.2573	−0.9720	−0.9719	−0.8212
RAI	0.9694	−0.9597	0.0894	0.8326	0.9937	0.9670
I_λ_ (−12 °C)	−0.9875	0.9307	7.05 × 10^−5^	−0.8788	−0.9997	−0.9403
I_λ_ (−18 °C)	−0.9979	0.8261	0.2222	−0.9629	−0.9798	−0.8413

## Data Availability

Not applicable.
